# Pertussis in Infancy: A Case of Delayed Diagnosis

**DOI:** 10.7759/cureus.92322

**Published:** 2025-09-14

**Authors:** Azary Hernandez, Jean Paul Ambroise, Ana M Hernandez-Puga

**Affiliations:** 1 Internal Medicine, Jackson Memorial Hospital, Miami, USA; 2 Medicine, Jackson Memorial Hospital, Miami, USA; 3 Pediatrics, Baptist Health South Florida, Miami, USA

**Keywords:** dtap vaccine, late diagnosis, pertussis vaccination, vaccine hesitancy, vaccine-preventable diseases

## Abstract

*Bordetella pertussis* is a gram-negative bacterium that primarily infects the respiratory tract. We report the case of a 13-month-old male patient who developed pertussis. At the initial clinic presentation, the diagnosis was not immediately considered, as the early manifestations closely mimicked those of a viral upper respiratory infection. As the illness progressed, the classic paroxysmal cough with inspiratory “whoop” became apparent, prompting the correct diagnosis. At that time, a nasopharyngeal swab was obtained, which tested positive for *Bordetella pertussis* by polymerase chain reaction (PCR). The patient was prescribed a five-day course of azithromycin, and close contacts were given prophylactic treatment.

This case underscores the critical importance of early recognition and timely diagnosis to prevent potentially life-threatening complications. It also highlights the necessity of vaccination awareness. Educating parents and caregivers on the benefits of immunization against vaccine-preventable diseases is essential to safeguard vulnerable individuals and to promote community-wide protection through herd immunity.

## Introduction

Pertussis, or whooping cough, is a highly contagious respiratory infection caused by the gram-negative coccobacillus *Bordetella pertussis*. The bacterium adheres to the cilia lining the upper respiratory tract via virulence factors such as pertussis toxin (PT) and filamentous hemagglutinin (FHA) [1]. Pertussis toxin, a protein exotoxin, and filamentous toxin work together to bind to the ciliated cells in the respiratory epithelial cells [2]. Following attachment, a local peribronchial lymphoid hyperplasia develops, progressing to necrotizing inflammation and leukocytic infiltration of the larynx, trachea, and bronchi [2]. There have been no correlations that have been proven between the pertussis toxin and the paroxysmal coughing that is very prominent in infected pediatric patients. 

Although the toxin has not been proven to be directly linked with the paroxysmal cough, it is still a very prominent symptom in this disease. After exposure to the bacteria, there is an incubation period of five to 10 days for symptoms to develop [1]. In its early phase, pertussis presents with symptoms that closely mimic those of the common cold, often leading to delayed recognition and progression to respiratory distress [1]. Early symptoms, as previously stated, are similar to the common cold; patients will have a runny nose, mild cough, and a low-grade fever, all within the first two weeks. In certain cases, infants with pertussis may experience apnea resulting in cyanosis and significant respiratory compromise [1]. Later symptoms emerge after approximately two weeks and may persist for one to 10 weeks. During this stage, the patient develops a paroxysmal cough that may cause sleep disturbance, respiratory difficulty, a high-pitched inspiratory “whoop,” and post-tussive emesis [1]. Serious complications occur in approximately one-third of infants under one year of age. Among hospitalized patients in this age group, apnea occurs in two-thirds, pneumonia in one-fifth, convulsions in one-fifth of hospitalized children, encephalopathy in less than 1% of hospitalized children, and death in 1% of these children [1]. In adults and teens, complications like pneumonia can occur but are generally less severe, especially in those who are vaccinated.

As previously noted, early detection of pertussis is challenging due to its initial symptoms closely resembling those of a common cold. Additionally, because pertussis incidence has historically been low, largely attributable to widespread vaccination, both parents and clinicians may have a low index of suspicion, complicating timely diagnosis. However, recent surveillance data indicate an increase in cases; for example, in Florida, reported pertussis cases numbered 55, 60, and 85 in 2021, 2022, and 2023, respectively [3]. The gradual increase in cases each year reflects a true rise in incidence, rather than changes in testing or reporting. In 2024, reported cases increased sharply to 708, representing a sevenfold rise compared to the previous year. The majority of cases occurred in children under one year of age, the demographic most vulnerable to severe symptoms and complications [3]. Pertussis is a vaccine-preventable disease, and it is well established that even a single dose of the vaccine can reduce the severity of symptoms compared to unvaccinated individuals [3]. 

Vaccination for pertussis became available in the 1940s; children are vaccinated at ages two, four, and six months [4]. The diphtheria, tetanus, and acellular pertussis (DTaP) vaccine has been available since 1990 and contains diphtheria toxoid, tetanus toxoid, inactivated PT, FHA, pertactin, and fimbrial antigens [4]. Prior to 1990, the whole-cell pertussis vaccine, which contained the entire inactivated *Bordetella pertussis* bacterium, was used [4]. This vaccine was more effective at preventing infection compared to the current acellular vaccine [4]. However, the acellular vaccine remains effective in reducing symptom severity and preventing disease progression [4]. Children under one year of age are at the highest risk for severe pertussis infection. Therefore, preventing disease progression and associated complications is critical. As previously noted, serious complications in this age group may include apnea, pneumonia, seizures, encephalopathy, and death. Vaccines have contraindications; therefore, it is important to inform the vaccine provider of any history of life-threatening allergic reactions to previous vaccines or known allergies to any components of the vaccine [1]. Additionally, individuals should disclose any history of seizures, neurological disorders, Guillain-Barré syndrome, or severe pain and swelling following a previous tetanus or diphtheria vaccination [1]. Common side effects of the DTaP vaccine may include injection site swelling and soreness, fever, irritability, fatigue, decreased appetite, and vomiting [1]. The side effects of the tetanus, diphtheria, and pertussis (Tdap) vaccine, administered to adolescents and adults, may include pain, redness, and swelling at the injection site; mild fever; headache; fatigue; nausea; vomiting; diarrhea; and abdominal pain [1].

## Case presentation

The patient was a 13-month-old male toddler who came into the clinic on May 12, 2025, for a cough of three to four days. His parents stated that when he coughed, it sounded like he was trying to get something out, but would choke. He turned blue during bouts of coughing. That morning, the patient coughed so hard after breakfast that he threw up his food. No fever was noted, but he did feel warm to the touch. Parents stated that the cough was worse at night. The patient was still eating and playing fine, but was low in energy and did not want to walk. The initial diagnosis was upper respiratory tract infection, treated with nebulized saline, cetirizine (Zyrtec), and albuterol sulfate nebulization at 2.5 mg/3 mL (0.083% solution), 3 mL every four to six hours as needed for bronchospasm. On May 15, the patient was brought into the clinic again by his parents because of a worsening cough. The patient had a prolonged paroxysmal cough leading to apnea and cyanosis (blue/purple discoloration). Caregivers reported breath-holding episodes lasting longer after food intake and at night. The patient remained afebrile and interactive between episodes. 

Vaccination history included the first dose of measles, mumps, and rubella (MMR) vaccine administered on April 23, 2025, complicated by rash development, and completion of the first three doses of DTaP. The patient was only 13 months old at the time of this clinic visit, and he had been given the CDC-recommended doses of DTaP at two, four, and six months of age. A physical exam revealed erythema and effusion of the left tympanic membrane and diffuse wheezing on lung auscultation. A nasopharyngeal swab was obtained on May 15 at the outpatient clinic and was sent out. A positive polymerase chain reaction (PCR) for *Bordetella pertussis* came back on May 17, as seen in Table 1. On May 15, when all clinical presentations and in-office paroxysmal cough that led to cyanosis were observed, the patient was given his treatment, which included azithromycin suspension dosed at 200 mg/5 mL, 3 mL orally on day 1, followed by 1.5 mL once daily for four additional days (total 15 mL over five days). Household contacts and medical staff who had contact with the patient were also prescribed azithromycin for prophylaxis on May 17 when the positive PCR for *Bordetella pertussis* came back. While azithromycin does not significantly alter symptoms in later stages, early administration is essential to reduce transmission risk and improve prognosis, which in this case is favorable with antibiotic therapy [5].

**Table 1:** Results from the sample obtained on May 15, 2025 On May 15, in-clinic testing for influenza A and B, as well as a rapid streptococcal antigen test, returned negative results. A polymerase chain reaction (PCR) sample for *Bordetella pertussis* was subsequently sent, which returned positive on May 17, confirming the diagnosis.

Labs	Results	Normal value
Rapid Streptococcus antigen test	Negative (in office)	Negative
Influenza A	Negative (in office)	Negative
Influenza B	Negative (in office)	Negative
Pertussis PCR	Positive (sent out)	Negative

## Discussion

Pertussis is an infection that has early symptoms that can be easily confused with other respiratory diseases. This is a contributing factor to why whooping cough might be difficult to diagnose at first, and when misdiagnosed early on, it allows the infection to progress. The progression can lead to serious complications as previously stated. Misdiagnosis occurs more frequently than expected, largely due to the overlap of clinical symptoms with other respiratory infections. Some conditions that could mimic pertussis include pneumonia (*Mycoplasma pneumoniae*, *Chlamydophila pneumoniae*, etc.), respiratory syncytial virus (RSV), upper respiratory tract infections, parainfluenza, common cold, influenza, asthma, etc. [6]. In this case, there were some key diagnostic features that raised suspicion for pertussis. The patient presented with paroxysmal cough with cyanosis and vomiting, prolonged breath-holding spells, and times when everything was normal [7]. All of these presentations should be taken into consideration when diagnosing. 

Pertussis should be considered even if patients are partially vaccinated. In this case, the patient received three doses of DTaP, which granted partial immunity. A booster dose is needed for full protection. It is important that the public is aware of the scheduling and compliance of vaccine administration. The current guidelines by the CDC are crucial for protecting individuals and communities from diseases [8]. Vaccine hesitancy has been an emerging problem in the healthcare system. Some of the causes of this problem include lack of confidence in vaccines, misinformation, fear of side effects, media coverage, local beliefs, political views/beliefs, influences (medical, political, or cultural), etc. [9,10]. Although vaccines will not fully stop a person from getting infected, they will protect them from severe symptoms and complications. This is especially important for the age group of children under a year of age, who are more susceptible to the worst complications. Some consequences of vaccine hesitancy can include lower vaccination rates, increased spread of infections, hospitalizations, and death. Currently, there are solutions to decrease this emerging wave of vaccine hesitancy. Effective strategies like public education, coercive techniques, combating misinformation online, etc., have proven to minimize vaccine hesitancy [11,12].

The combination of vaccine hesitancy, low immunization rates, and lack of trust in recent years has contributed to an increase in pertussis outbreaks. In 2025, there were more reported cases in the US compared to 2023 [13,3]. The CDC reports that the number of cases has increased over the last decade and believes that many cases have been unrecognized and unreported [14].

Early diagnosis and appropriate antibiotic therapy are effective ways to treat pertussis. The goal is to reduce transmission, especially in the infant/child population. In this case, the patient and close contacts were given azithromycin. Timing and supportive care play an important role in the treatment of pertussis. It is important to note that antibiotics, in this case or any case of pertussis, are less effective for symptom relief in the later stages of pertussis but are highly important for preventing spread. Current CDC guidelines advise that for symptom reduction and severity, it is best to treat within the first two weeks [15].

The relevance of this case is important because it shows that there is a challenge when diagnosing pertussis in a partially vaccinated infant patient with abnormal symptoms that were mistaken for an upper respiratory infection. This case highlights that pertussis, even when vaccinated, can still be a challenge to recognize, diagnose, and treat. Healthcare professionals should stay alert for pertussis even if patients have been immunized. As seen in this case, even if classic presentations occur, they can still be misdiagnosed as other respiratory infections. CDC guidelines state the best methods for diagnosing pertussis are culture (gold standard), PCR, or serology [15]. As mentioned before, treatment for this disease depends highly on the timing. Later stages of pertussis may not resolve symptoms, but are essential in the prevention of spreading. Also, treatment of close contacts, like in this case, plays an important role in decreasing community transmission. As seen in this case, healthcare providers should consider and test for pertussis earlier with the classic symptoms, even if vaccination is not complete. This case highlights the importance of full vaccinations against pertussis and reinforces the role of public health measures.

## Conclusions

Although vaccines may not always prevent infection, they still protect immunocompromised children under the age of one from developing life-threatening complications. This case emphasizes the importance of childhood vaccination. Prevention of infection and complications is possible by increasing awareness among parents and caregivers about the significance of vaccination. Additionally, healthcare professionals should educate families and advocate for adherence to vaccination schedules. To reduce spread and limit complications, it is important to recognize the disease early and initiate treatment following established guidelines. Further research and public health efforts are needed to improve vaccination coverage and public awareness.
